# An effective co-modification strategy to enhance the cycle stability of LiNi_0.8_Co_0.1_Mn_0.1_O_2_ for lithium-ion batteries†

**DOI:** 10.1039/d3ra04145j

**Published:** 2023-11-22

**Authors:** Jingjing Zhou, Bingxin Wei, Meng Liu, Yinping Qin, Hongyu Cheng, Yingchun Lyu, Yang Liu, Bingkun Guo

**Affiliations:** a Materials Genome Institute, Shanghai University, Shanghai 99 Shangda Road Baoshan District Shanghai P. R. China liuyang81@shu.edu.cn guobingkun@shu.edu.cn; b Wuhan Institute of Marine Electric Propulsion Wuhan 430064 P. R. China; c A Key Laboratory of Advanced Energy Materials Chemistry (Ministry of Education), Nankai University Tianjin 300071 P. R. China; d Key Laboratory of Optoelectronic Chemical Materials and Devices of Ministry of Education, Jianghan University No. 8, Sanjiaohu Rd. Wuhan Hubei 430056 P. R. China

## Abstract

Ni-rich cathode materials suffer from rapid capacity fading caused by interface side reactions and bulk structure degradation. Previous studies show that Co is conducive to bulk structure stability and sulfate can react with the residual lithium (LiOH and Li_2_CO_3_) on the surface of Ni-rich cathode materials and form a uniform coating to suppress the side reactions between the cathode and electrolyte. Here, CoSO_4_ is utilized as a modifier for LiNi_0.8_Co_0.1_Mn_0.1_O_2_ (NCM811) cathode materials. It reacts with the residual lithium on the surface of the NCM811 cathode to form Li-ion conductive Li_2_SO_4_ protective layers and Co doping simultaneously during the high-temperature sintering process, which can suppress the side reactions between the Ni-rich cathode and electrolyte and effectively prevent the structural transformation. As a result, the co-modified NCM811 cathode with 3 wt% CoSO_4_ exhibits an improved cycling performance of 81.1% capacity retention after 200 cycles at 1C and delivers an excellent rate performance at 5C of 187.4 mA h g^−1^, which is 10.2% higher than that of the pristine NCM811 cathode.

## Abstract 2

A co-modified NCM811 cathode material with Co doping and a Li_2_SO_4_ coating is obtained by reaction of CoSO_4_ modifier with residual lithium *via* a one-step facile method.

## Graphical abstract

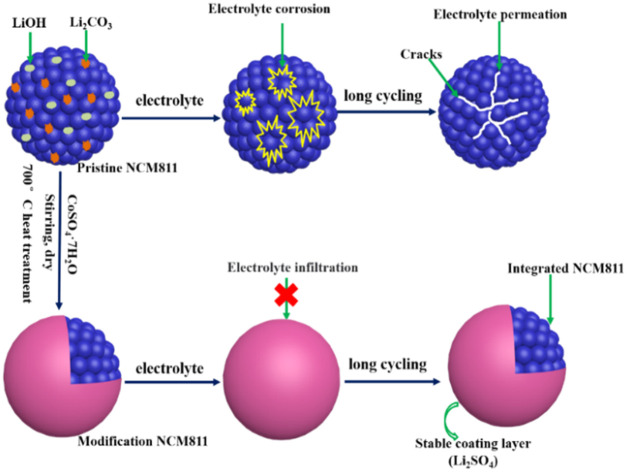

## 1 Introduction

Among the state-of-the-art electrochemical energy storage devices, lithium-ion batteries (LIBs) are the most promising and world-changing energy storage devices, as they possess high energy density and fast recharging capability. In order to meet the ever-increasing desire for high energy density, Ni-rich ternary cathode materials have become the most promising candidates.^1–4^

However, they are still facing the shortcoming of rapid capacity fading, which limit their practical application.^5^ These drawbacks are caused by the following: Firstly, during cycling, the anisotropic structure transformation leads to the structure collapse, and the rock-salt type disordered surface structures with a higher activation energy barrier for Li^+^ migration decrease the electrochemical performance.^6,7^ Secondly, during the charging process, Ni^2+^ and Ni^3+^ oxidize to Ni^4+^, which can react much more easily with the electrolyte by parasitic reactions.^8,9^ Thirdly, the residual lithium on the surface of the material is easy to react with H_2_O and CO_2_ in the air to form a Li_2_CO_3_/LiOH passivation layer, which not only increases the interface impedance, but also leads to an irreversible capacity loss during the first cycle.^10–12^

To address the above issues, numerous methods, such as surface coating and element doping, are widely used to enhance the interface or bulk stability of Ni-rich cathode materials.^13^ Doping with a metallic element (Ti, Mg, Al, Co *etc*) into the bulk crystallographic structure of Ni-rich materials is considered to be a representative method to strengthen the structural stability of the whole particle.^14–18^ Zhang *et al.* reported that Ti^4+^-doped Ni-rich cathode LiNi_0.8_Co_0.1_Mn_0.1_O_2_ shows multiple performances with high capacity, good rate performance and excellent cyclic stability.^14^ Sun *et al.* reported that Mg and Al co-substituted LiNi_0.8_Co_0.1_Mn_0.1_O_2_ have positive effects on structure, electrochemical property and thermal stability.^15^ Guo *et al.* found that Co doped LiNi_0.8_Co_0.1_Mn_0.1_O_2_ cathode material shows high capacity and maintain notable cycling stability in 300 cycles.^17^ Based on first-principle calculation, Hu *et al.* speculated that Co doping can increase the space distance of the Li layer, which is beneficial to improve the rate performance of Ni-rich cathode.^18^ Also, surface coating of Ni-rich materials by metal oxides (Al_2_O_3_, TiO_2_, ZrO_2_),^19–21^ metal fluorides (LiF, LaF_3_)^22,23^ and metal phosphates (Li_3_PO_4_)^24^ also has been widely reported. Lithium-ion conductor can enhance the surface chemical and structural stability, by acting as a protective layer that avoids direct reactions between the active materials and the electrolyte components.^25^ Aurbach *et al.* reported that Li_2_SO_4_ coating layer can suppress the side reaction between Ni-rich cathode and electrolyte due to its high stability at high voltage.^26^ Although these coatings can minimize direct contact between the highly active cathode and the electrolyte and therefore alleviate parasitic reactions, reduce impedance growth, and suppress microcrack in some extent, most coatings are difficult to make a bonding with the bulk, and unable to afford a long-term effective protection. A functional coating which can afford close integration between coating layer and bulk materials is the promising option. It is reported that taking advantage of a modifier which can react with the residual lithium (LiOH and Li_2_CO_3_) on the surface of LiNi_0.8_Co_0.1_Mn_0.1_O_2_ cathode material and forming a uniform coating is an ideal way to make the close integration between coating layer and bulk materials.^27^

**Scheme 1:**
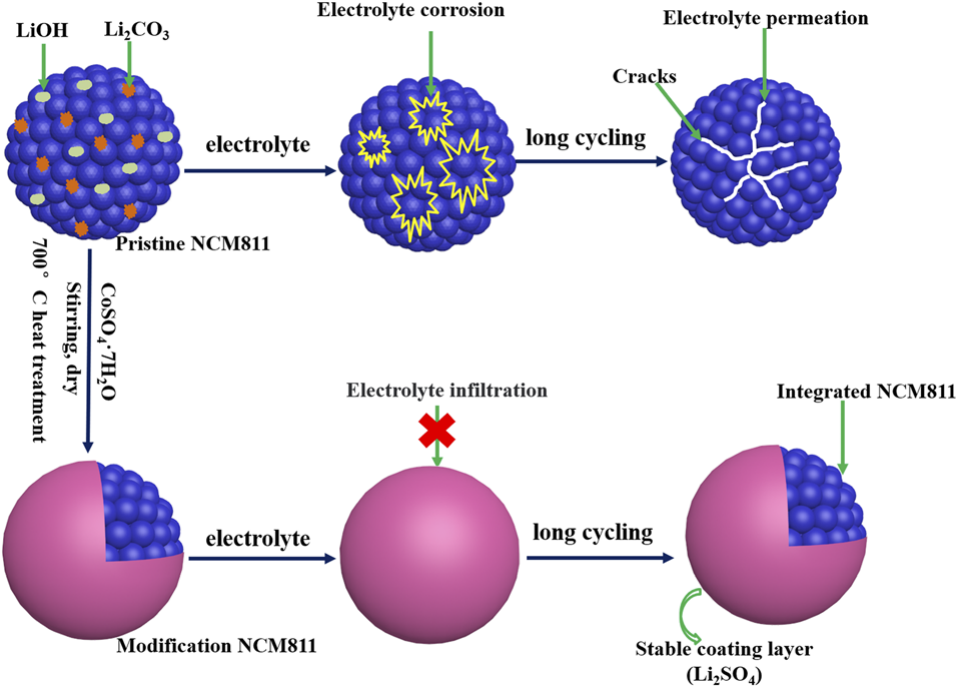
Illustration of modified NCM811 preparation and the evolution of particles after long cycling.

Based on the above considerations, a simple and effective method was proposed to modify LiNi_0.8_Co_0.1_Mn_0.1_O_2_ (NCM811) from surface to bulk. Co-modified NCM811 cathode materials with different content CoSO_4_ as modifiers are prepared, which combine Co doping and Li_2_SO_4_ surface coating with removing residual lithium *via* a one-step facile method, as shown in Scheme 1. The influence of co-modified on the physical structure and electrochemical properties of NCM811 cathode materials were explored. The co-modified NCM811 cathode with 3 wt% CoSO_4_ exhibits an improved cyclic stability and rate performance. The modification removes residual lithium and suppresses the side reactions between cathode materials and electrolyte, improves the surface and bulk structure stability and promotes Li^+^ ion diffusion simultaneously.

## 2 Methods

### 2.1 Preparation

The spherical precursor was prepared by the co-precipitation method as described previously.^28^ The obtained Ni_0.8_Co_0.1_Mn_0.1_(OH)_2_ precursor was mixed with LiOH·H_2_O at the molar ratio Li : TM = 1.05 : 1, the mixture was calcination at 800 °C for 10 h with flowing O_2_, abbreviated as NCM811.

CoSO_4_-modified NCM811 was prepared by mixing CoSO_4_·7H_2_O and NCM811 under mortaring for 30 min. The mixtures were calcined at 600, 700, 800 °C for 4 h with flowing O_2_. The content of CoSO_4_·7H_2_O in the mixture was 1, 3 and 5 wt%, and the obtained samples were labeled as NCM811-CS-1, NCM811-CS-3, and NCM811-CS-5. The equivalent molar amount of CoC_2_O_4_·2H_2_O and Li_2_SO_4_ as modifiers were replaced of 3 wt% CoSO_4_ to modify NCM811 respectively. Li_2_SO_4_-modified NCM811 and CoC_2_O_4_-modified NCM811 were prepared under the same condition of the NCM811-CS-3, which denoted as NCM811-Li_2_SO_4_ and NCM811-CoC_2_O_4_ respectively.

### 2.2 Characterization

The morphology and the elements distribution of the prepared samples were characterized using scanning electron microscopy (SEM, Hitachi Regulus 8230). X-ray-diffraction (XRD) patterns of all samples was characterized using PANalytical Empyrean, from 10° to 90°, with Cu Kα (*λ* = 1.5406 Å) radiation.

The elemental analysis of pristine NCM811 and NCM811-CS-3 were investigated by inductively coupled plasma optical emission spectrometry (ICP-OES, Agilent 5110) and inductively coupled plasma mass spectrometry (ICP-MS, Agilent 7800). The components Li, Mn, Ni, and Co were measured by ICP-OES and S was measured by ICP-MS. The compositions of pristine and modified NCM811 electrodes were characterized using an ESCALAB 250Xi X-ray photoelectron spectrometer aided with Ar^+^ ion etching.

### 2.3 Electrochemical testing

Electrochemical performance was measured by CR2032 coin-type cells. Cathodes were prepared by 80 wt% of active material, 10 wt% of Super-p and 10 wt% of poly(vinylidenefluoride) (PVDF), which were mixed with *N*-methyl-2-pyrrolidone (NMP) as the slurry, and then taking the slurry to coat on an Al current collector, and roll-pressed. The cells were assembled using lithium metal as the anode, microporous membranes (Celgard 2550s) as a separator, 1 mol L^−1^ LiPF_6_ in a mixed solution containing ethylene carbonate (EC)/dimethyl carbonate (DMC)/ethyl methyl carbonate (EMC) with volume ratio of 1 : 1 : 1 as electrolyte. All cell assemblies were performed in an Ar-filled MBraun glovebox, where humidity and oxygen were controlled to less than 1 ppm. The cycling performance, rate capability and galvanostatic intermittent titration technique (GITT) test were performed using a LANHE CT2001A battery tester (Wuhan, China) at room temperature under various rates. 1C rate corresponds to a current density of 200 mA h g^−1^ in this work. The duration time for each applied galvanostatic current (0.1C) and relaxation during the GITT measurements was 0.5 and 4 h, respectively. Electrochemical impedance spectroscopy (EIS) was measured on a Solartron analytical 1470 Cell Test System in a frequency range of 0.1–10^5^ Hz. And conductivities of pristine and modified NCM811 materials under different pressures were also tested on the Solartron Cell Test System.

## 3 Results and discussion

The different temperatures of 600, 700 and 800 °C for modification of NCM811 are investigated. As shown in Fig. S1,† the cycling performance of the sample calcined at 700 °C is the best. And the annealing of 700 °C for the electrochemical performance of pristine NCM811 was also studied shown in Fig. S2.† The extra annealing shows no difference for the samples, thus 700 °C was selected for the modified temperature in this manuscript.

**Fig. 1:**
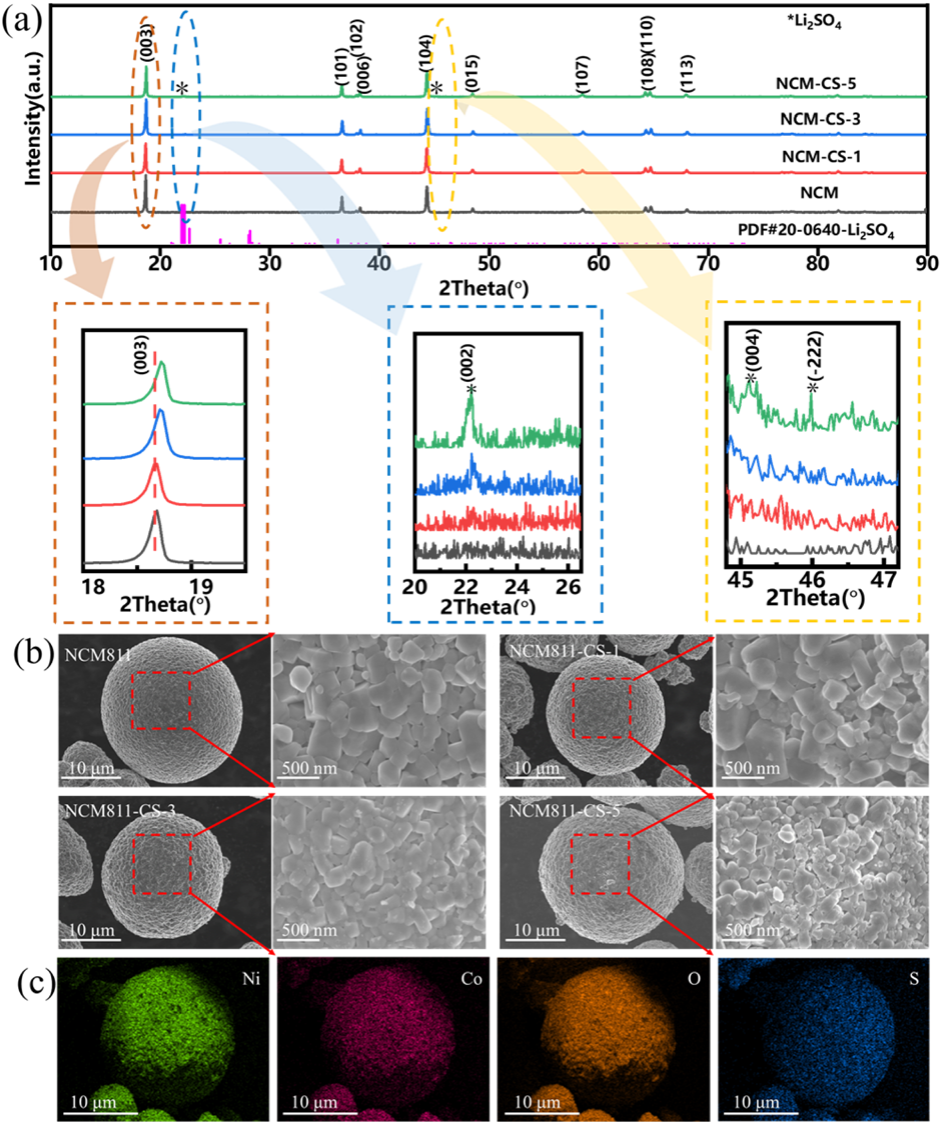
(a) XRD patterns of NCM811 and NCM811-CS; (b) SEM image of NCM811 and NCM811-CS; (c) EDS mapping of Ni, Co, O and S elements of NCM811-CS-3.

The XRD patterns of pristine and modified NCM811 materials have been shown in Fig. 1a, which are well-indexed as the hexagonal α-NaFeO_2_ layered structure with the space group *R*-3*m*.^29^ All samples show marked splitting peaks of (006)/(102) and (108)/(110), illustrating the well-ordered layered structure. With the increase of CoSO_4_ content, the additional diffraction peaks belonging to monoclinic Li_2_SO_4_ with space group *P*2(1)/*c*, at 2*θ* = 21–26°& 44–47°as marked by asterisks.^26^ It suggests a surface coating layer of Li_2_SO_4_, resulting from the reaction of CoSO_4_ and the lithium residue. Li_2_SO_4_ is regarded as a lithium-ion conductor, which is beneficial for the ion diffusion at the surface. In addition, the (003) peak shifts to higher diffraction angle with the increasing CoSO_4_ content. The lattice parameters have been refined by Rietveld method as shown in Fig. S3 and Table S1.† Results show that the *a*, *c* lattice parameters increase with the percentage of CoSO_4_·7H_2_O treatment. These indicate that Co^3+^ ions are doped into the bulk structure and the composition of NCM811-CS-3 is Li(Ni_0.8_Co_0.1_Mn_0.1_)_0.99_Co_0.01_O_2_ as the molar amount of doping Co is 0.01.

The elemental analyses of the pristine NCM811 and NCM811-CS-3 are performed using ICP-OES and ICP-MS technique. As indicated in Table S3,† components Li, Mn, Ni, and Co are detected for the above two samples, and S is detected for NCM811-CS-3. The atom ratios of Li, Ni, Mn are almost the same for NCM811 and NCM811-CS-3, while Co and S increases. The increment of Co and S is consistent with the molar quantity of 3 wt% CoSO_4_·7H_2_O in NCM811-CS-3.

The SEM images of pristine and modified NCM811 materials have been shown in Fig. 1b, which demonstrate that all samples possess spherical secondary particles with an average size of 10 μm comprising submicron primary particles, indicating that the co-modification treatment presents little effect on particle size, but the surfaces became rough as the CoSO_4_ content increasing. As shown in Fig. 1c, the EDS mapping results show that the S element is homogeneously distributed on the surface of the modified sample. XPS measurement aided with Ar^+^ ion etching also reveal the Li_2_SO_4_ on the surface of modified NCM811 and the thickness of the Li_2_SO_4_ coating layer is no more than 50 nm (Fig. S4 and S9†). These confirm that a uniform and thin Li_2_SO_4_ coating layer is formed on the surface of the NCM811 particles, in consistent with the XRD results.

**Fig. 2:**
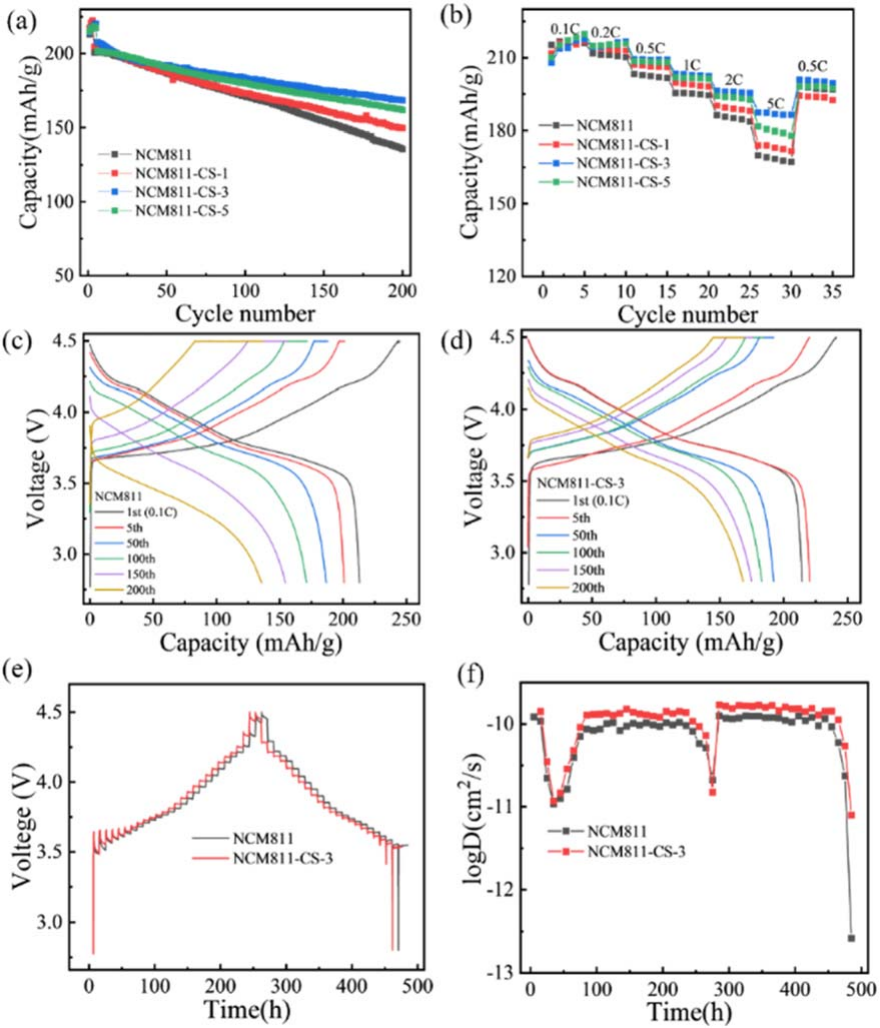
(a) Cycling performance of the cells cycled at 1C, (b) rate performance of the cells at various rates, charge/discharge voltage profiles of (c) NCM811 and (d) NCM811-CS-3 at various cycles; (e) GITT curves for NCM811 and NCM811-CS-3, (f) Li^+^ diffusion coefficient for NCM811 and NCM811-CS-3.

The electrochemical performance of pristine and modified NCM811 materials were tested between 2.8 and 4.5 V *vs.* Li^+^/Li. As shown in Fig. 2a, the first discharge capacities of NCM811 & NCM811-CS-3 are 213.5 and 214.8 mA h g^−1^. The plotted d*Q*/d*V* profiles show that the phase transitions of NCM811-CS-3 is similar with NCM811, but a low potential of the main oxidation peak, demonstrating a smaller polarization of NCM811-CS-3 (Fig. S5†). The pristine NCM811 displays rapidly capacity attenuation from 200.7 mA h g^−1^ to 135.6 mA h g^−1^ with 67.6% capacity retention in 200 cycles at 1C. In contrast, NCM811-CS-1, NCM811-CS-3, and NCM811-CS-5 exhibit higher capacity retention of 73.0%, 81.1% and 80.3%, respectively. Obviously, the NCM811-CS-3 exhibits the superior cycling stability. Fig. 2c and d show the evolution of charge/discharge profiles of NCM811 and NCM811-CS-3. NCM811 shows a rapid decline in the capacity, while few changes are observed in the discharge capacities and voltage platform of NCM811-CS-3. This demonstrates that the Co^3+^ doping with Li_2_SO_4_ coating for the NCM811 electrode can effectively relieve capacity fading.

To investigate the Co^3+^ doping and Li_2_SO_4_ layer coating by two separate processes on electrochemical performance, the equivalent molar amount of CoC_2_O_4_ and Li_2_SO_4_ as modifiers were replaced of CoSO_4_ to modify NCM811 respectively. As shown in Fig. S6,† the electrochemical performance of NCM811-Li_2_SO_4_ and NCM811-CoC_2_O_4_ are worse than that of NCM811-CS-3, even that of the pristine NCM811. The reasons for these results were inferred as follows: the co-modified NCM811 cathode material in this manuscript is a one-step facile method by the chemical reactions of CoSO_4_ with residual lithium to form Co doping and Li_2_SO_4_ coating. The separate process of Co^3+^ doping and Li_2_SO_4_ layer coating cannot achieve the results obtained by this one-step method. On the one hand, the separate process cannot remove the residual lithium on the surface, on the other hand, the formed coating layer may be nonuniform.

To evaluate the rate capabilities of the co-modification samples, the samples are measured between 0.1C–5C. The modified samples present the rate capabilities of 174, 187.4 and 181.7 mA h g^−1^ for NCM811-CS-1, NCM811-CS-3, and NCM811-CS-5 at 5C. They are higher than 170 mA h g^−1^ of the pristine NCM811 sample as shown in Fig. 2b. To gain a better understanding of the improved rate capabilities, the galvanostatic intermittent titration technique (GITT) was determined with a titration current of 0.1C, and the chemical diffusion coefficients are calculated,^25^ as shown in Fig. 2e and f. The overall trends of NCM811 and NCM811-CS-3 are almost the same, but NCM811-CS-3 delivers a higher Li^+^ diffusion coefficients than the pristine NCM811, validating that the co-modification has a positive effect on the improvement of Li^+^ diffusion kinetics. These improvements in electrochemical performance could be attributed to the both presence of Li_2_SO_4_ coating layer and Co-doping. The Li_2_SO_4_ ion conductor layer is benefit for the ion conductivity and the Co^3+^ doping is benefit for electronic conductivity.^30^ At the same time, they would improve the stability of the interface and bulk structure simultaneously.

The conductivities of pristine and modified NCM811 materials under different pressures were tested. As shown in Fig. S7,† the conductivities of all the samples increase with the pressure, and the conductivity of NCM811 is relatively higher than that of other modified materials. Since the electronic conductivity of NCM811 is much higher than its ionic conductivity, these results should be attributed to the surface electronic conductivity. According to these results, the Li_2_SO_4_ coating layer slightly reduces the surface electronic conductivity of NCM811. Combined with the results of GITT, the ionic conductivity of NCM811 after modified is improved. These demonstrate though the surface electronic conductivity of the modified material is reduced, the ionic conductivity is improved.

**Fig. 3:**
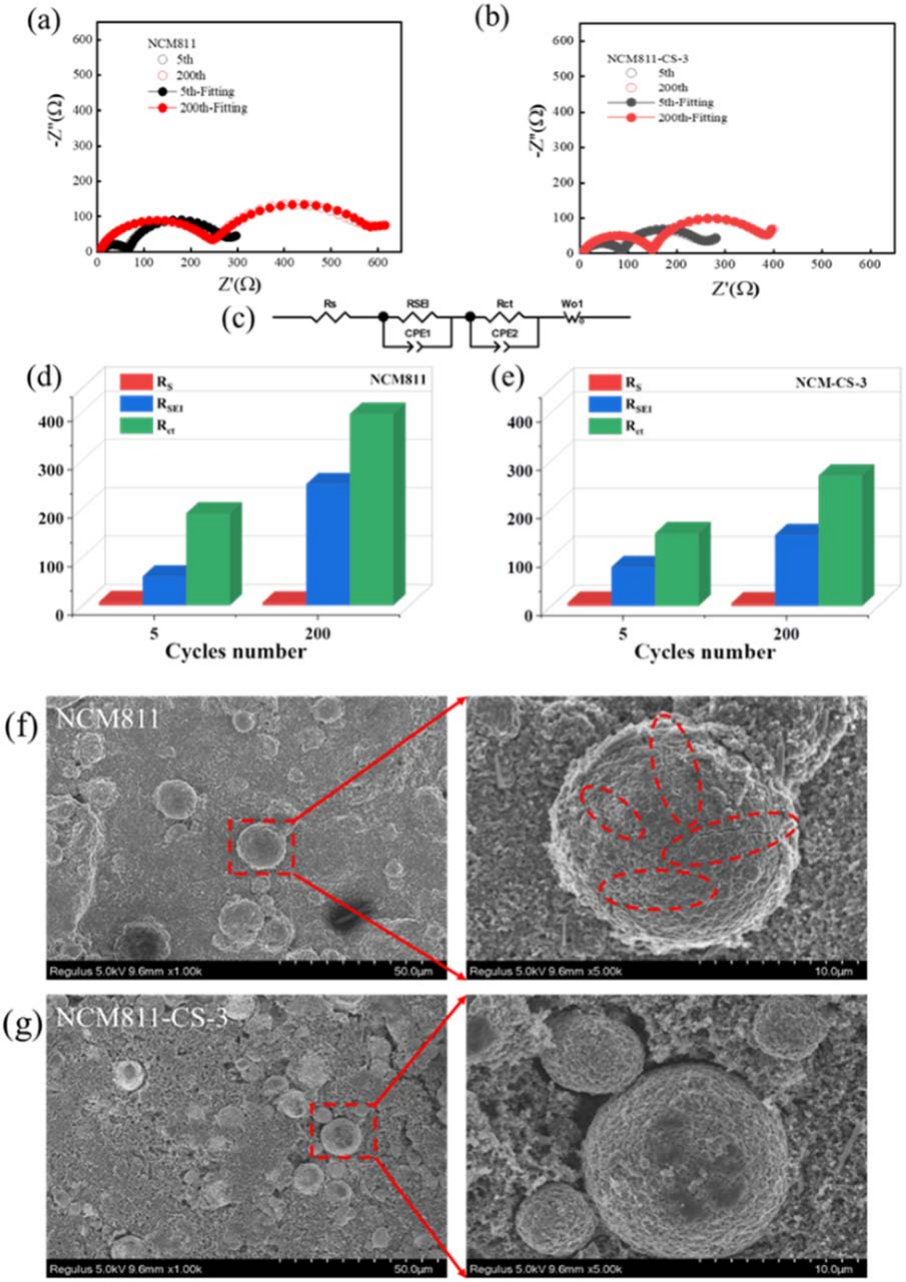
Nyquist plots of (a) NCM811 and (b) NCM811-CS-3 during cycling, (c) the equivalent circuit model, Fitted *R*_S_, *R*_SEI_ and *R*_ct_ value of (d) NCM811 and (e) NCM811-CS-3, SEM images of (f) NCM811 and (g) NCM811-CS-3 electrode after 200 cycles.

The electrochemical impedance spectroscopy (EIS) measurements were taken to investigate the impedance (Fig. 3a–e). The quantitative analysis was performed by fitting the spectra with an equivalent circuit model and the fitting results are shown in Fig. 3d and e. The resistances at high-frequency (first semicircle) and low-frequency (second semicircle) semicircles are derived from ion migration in interfacial film and charge transfer on electrodes, respectively, denoted as *R*_SEI_ & *R*_ct_. The *R*_SEI_ resistance of NCM811-CS-3 is slightly bigger than that of NCM811 in the 5th cycle, which should be attributed to the coating layer of Li_2_SO_4_. Although the presence of Li_2_SO_4_ coating increases the initial interface resistance of NCM811, the *R*_SEI_ resistance of NCM811-CS-3 is much lower than that of NCM811 in the 200th cycle. These indicate Li_2_SO_4_ coating layer improve the stability of NCM811 in the long-term cycling.

The *R*_ct_ resistance of NCM811-CS-3 is much lower than that of NCM811 in the 5th cycle, which should be related to good electronic conductivity of Co^3+^ doping. And the increase of *R*_ct_ resistance of NCM811 is much larger than that of NCM811-CS-3 in 200 cycles, suggesting that the co-modification improve the stability of the bulk structure. Therefore, though the initial *R*_SEI_ resistance of NCM811-CS-3 is slightly higher, the *R*_ct_ resistance is much lower, and the *R*_SEI_ & *R*_ct_ resistances are much more stable in the long-term cycling than those of NCM811. These suggest that co-modification improve the stability of the interface and bulk structure simultaneously.

Aiming at verifying the positive effect of the co-modification strategy, SEM images of the pristine NCM811 and NCM811-CS-3 after 200 cycles at 1C were obtained as shown in Fig. 3f and g. The pristine NCM811 particles suffer serious damage, where various number of cracks were observed due to volume anisotropic changes caused by phase transitions. Cracks would create new active surface and result in the continuous electrolyte decomposition, growth of the CEI, and poor cycling performance. In comparison, particle morphologies were well retained for NCM811-CS-3, further indicating the benefits of dual modification for NCM811 can maintain structural stability with reduced side reactions during cycling.

The structural variations of NCM811 and NCM811-CS-3 were compared before and after 200 cycles by XRD measurement. As shown in Fig. S8,† the shift of (003) peak of NCM811-CS-3 is smaller than that of NCM811 after 200 cycles. These indicate the variation of bulk structure of NCM811 is much larger than that of modified NCM811 after cycling. Therefore, the co-modification is conducive to the structural stability of NCM811 in the long cycling. XPS measurement of pristine and modified NCM811 displays that the S–O (169.5 eV) peak in S 2p is still strong on the surface of NCM811-CS-3 after 200 cycles (Fig. S9 and Table S2†). These indicate the Li_2_SO_4_ coating layer displays good stability at the high voltage of 4.5 V *vs.* Li^+^/Li for long cycling. In addition, EDS was used to test the content of transition metal elements deposited on lithium metal surface of pristine and modified NCM811 after 200 cycles. As shown in Fig. S10,† the dissolution of Mn and Ni elements were detected on the lithium metal side of NCM811 after 200 cycles, which were not be detected of the modified NCM811, indicating that co-modification can inhibit the dissolution of transition metals during the long-term cycling.

## 4 Conclusion

In summary, the co-modified LiNi_0.8_Co_0.1_Mn_0.1_O_2_ cathode materials with Li_2_SO_4_ coating and Co-doping were prepared by a facile and effective one-step strategy using CoSO_4_ as a modifier in this work. With adding 3 wt% CoSO_4_, NCM811-CS-3 shows the excellent cyclic stability (with 81.1% capacity retention in 200 cycles at 1C) and rate performance (187.4 mA h g^−1^ at 5C). The formation of Li_2_SO_4_ surface coating layer consumed the residual lithium compounds on the surface of Ni-rich cathode material and mitigated the side reactions between cathode materials and electrolyte. Meanwhile, the Co doping can improve the stability of the structure and promote Li^+^ ion diffusion. Thus, co-modified Ni-rich cathode materials deliver an excellent electrochemical performance. And the results indicate that this co-modification strategy can effectively improve the electrochemical performance. And the results indicate that this co-modification strategy can also be expected to apply to other oxide cathode materials.

## Conflicts of interest

There are no conflicts to declare.

## Supplementary Material

RA-013-D3RA04145J-s001
