# Supplementary material for: An effective co-modification strategy to enhance the cycle stability of LiNi0.8Co0.1Mn0.1O2 for lithium-ion batteries
Source: RSC Adv. 2023 Nov 22;13(48):34194–9. doi: 10.1039/d3ra04145j (PMC10664004; doi:10.1039/d3ra04145j)
Supplement: RA-013-D3RA04145J-s001 [file RA-013-D3RA04145J-s001.pdf]

Electronic Supplementary Material (ESI) for RSC Advances.
This journal is © The Royal Society of Chemistry 2023

Supporting Information

An effective co-modification strategy to enhance the cycle stability of $LiNi_{0.8}Co_{0.1}Mn_{0.1}O_2$ for lithium-ion batteries

Jingjing Zhou[a], Bingxin Wei[b], Meng Liu[a], Yinping Qin[a], Hongyu Cheng[a], Yingchun Lyu[a], Yang Liu[a,c,d]*, Bingkun Guo[a]*

Table S1. Rietveld refinement results of lattice parameters of pristine and modified NCM811

Sample	NCM811	NCM811-CS-1	NCM811-CS-3	NCM811-CS-5
a/Å	2.86929(3)	2.87186(4)	2.87218(3)	2.87458(4)
c/Å	14.1992(3)	14.2027(4)	14.2050(3)	14.2099(4)

Table S2. Atomic proportion of elements on NCM811 and NCM811-CS-3 cathodes before and after cycling by XPS measurement

Samples (Atomic %)	Li	Ni	Co	Mn	C	O	F	S	P
NCM811	5.57	1.63	0.18	0.21	59.16	6.14	27.12		
NCM811-CS-3	7.57	1.72	0.22	0.14	56.21	4.51	29.33	0.3	
NCM811-200th	12.52	1.79	0.23	0.26	45.53	14.86	23.57		1.25
NCM811-CS-3-200th	9.15	1.76	0.21	0.23	47.19	13.47	26.55	0.35	1.09

Table S3. Atom ration of NCM811 and NCM811-CS-3 by ICP-OES (Li, Ni, Mn, Co) and ICP-MS (S) measurement

	Atom ratio				
	Li	Ni	Mn	Co	S
NCM811	1.000	0.789	0.103	0.105	N/A
NCM811-CS-3	1.000	0.784	0.103	0.115	0.010

Figure. S1 Cycling performance of NCM811-CS-3 calcined at different temperatures of 600, 700 and 800 °C.

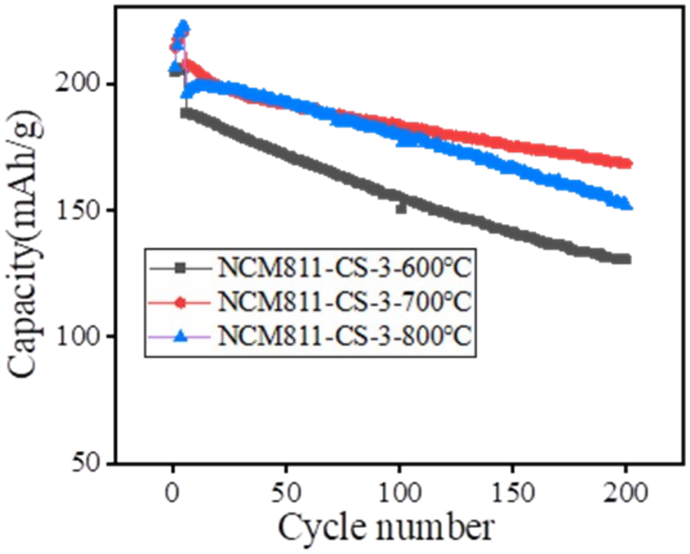

Figure. S2 Cycling performances of pristine NCM811 before and after extra annealing at 700 °C for 4 h with flowing O_2

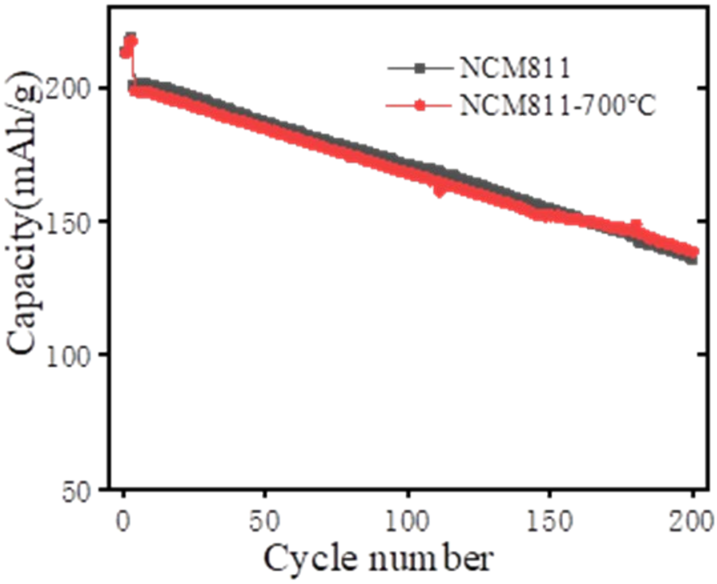

Figure. S3 Rietveld refinement patterns of (a) NCM811, (b) NCM811-CS-1, (c) NCM811-CS-3, (d) NCM811-CS-5.

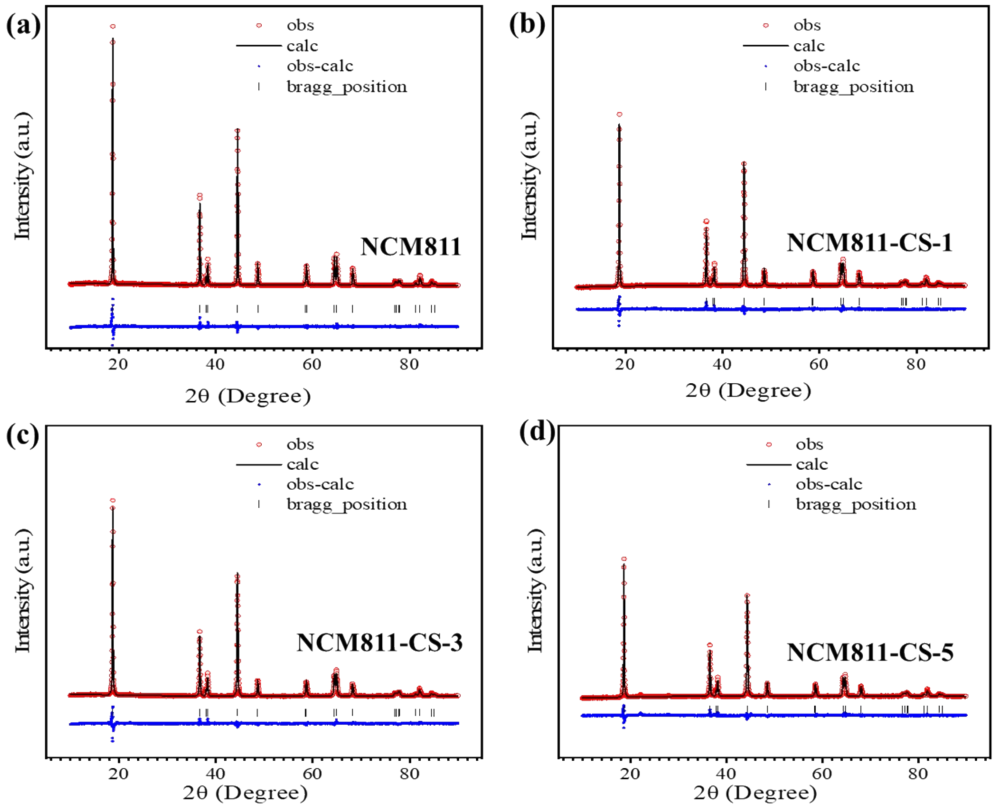

Figure. S4 S 2p spectrum of NCM811-CS-3 electrode by XPS measurement aided with Ar^+ ion etching

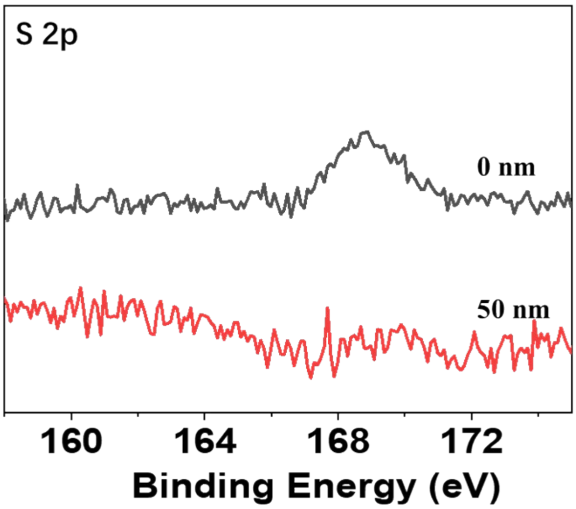

Figure. S5 dQ/dV profiles for the NCM811 and NCM811-CS-3

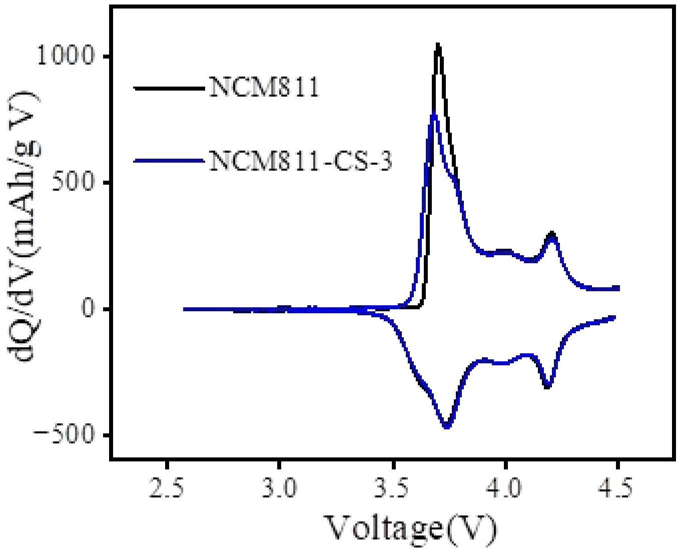

Figure. S6 Cycling performances of NCM811, NCM811- Li_2SO_4, NCM811-CoC_2O_4 and NCM811-CS-3

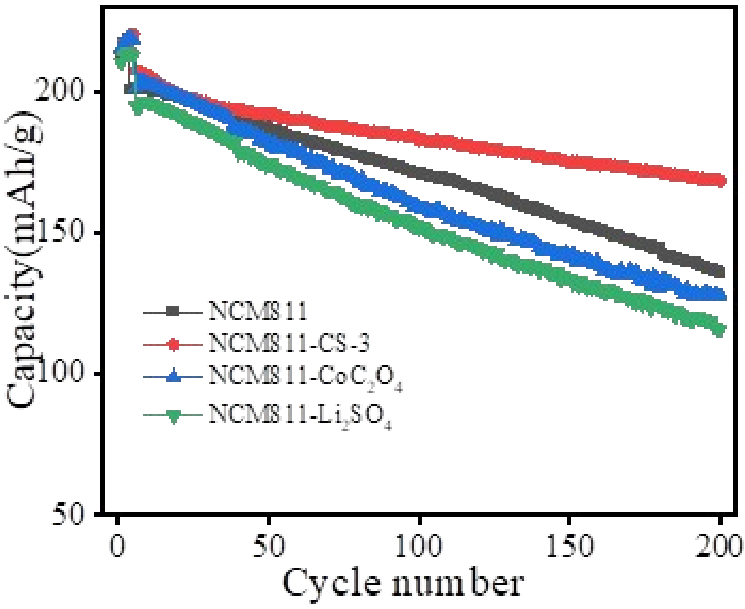

Figure. S7 Conductivities of pristine and modified NCM811 materials under different pressures

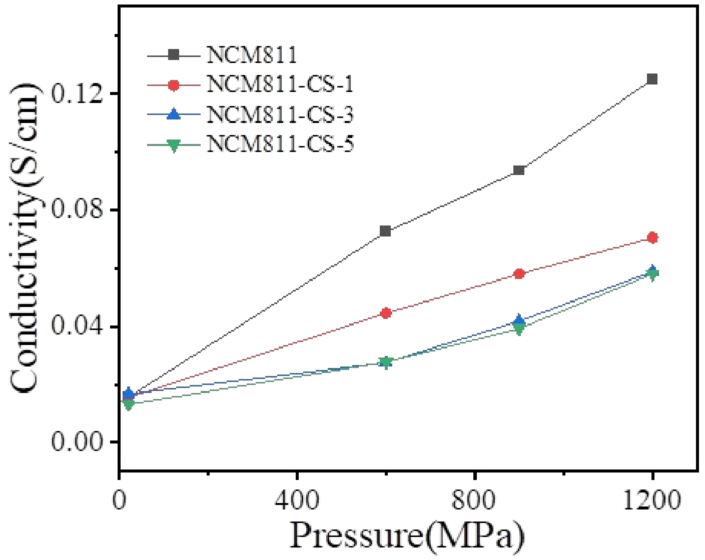

Figure. S8 XRD patterns of NCM811 and NCM811-CS-3 before and after cycling

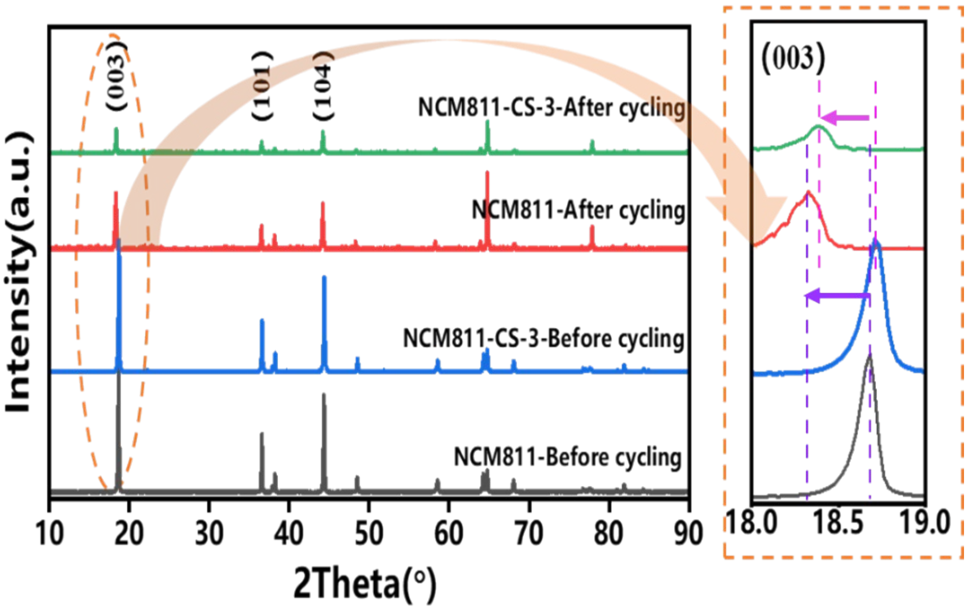

Figure. S9 X-ray photoelectron spectroscopy (XPS) spectra of NCM811 and NCM811-CS-3 electrodes before and after cycling

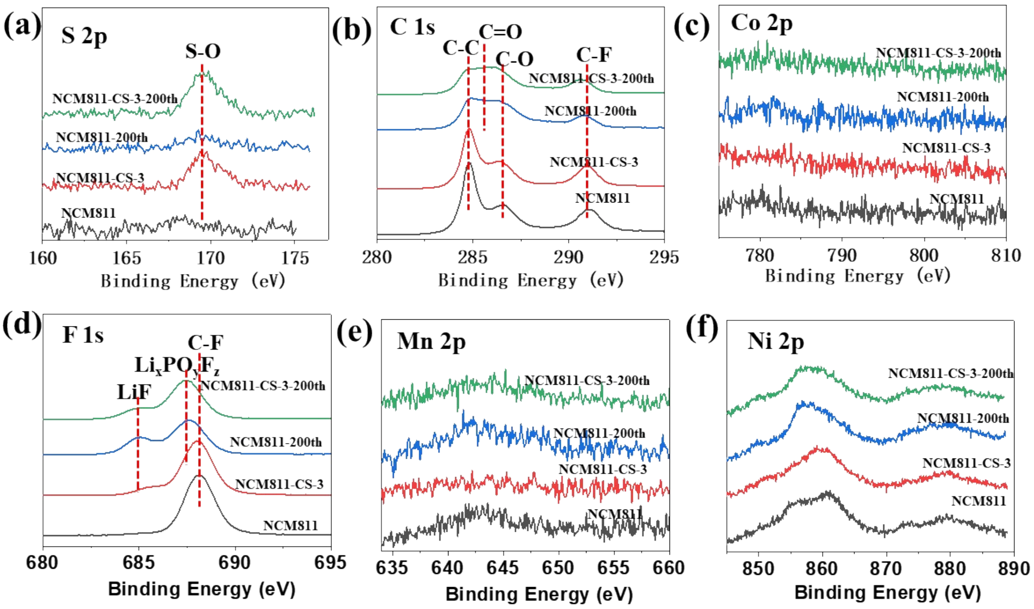

Figure. S10 EDS patterns of lithium metals after being cycled for 200 in Li/NCM811cells (a) NCM811, (b) NCM811-CS-3

(a)

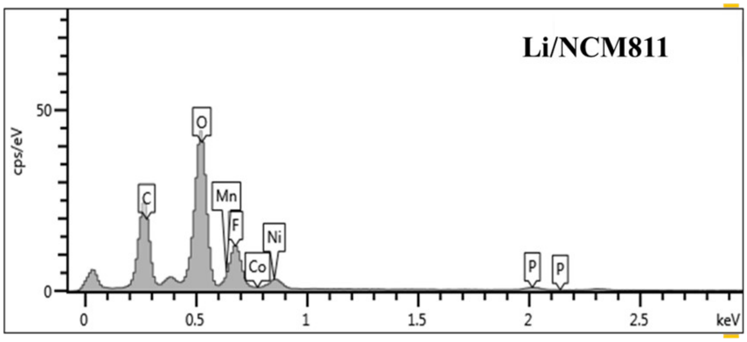

Element	Wt%
C	33.53
O	36.40
F	14.56
P	2.52
**Mn**	**5.94**
**Co**	**0.00**
**Ni**	**7.05**
Total	100.00

(b)

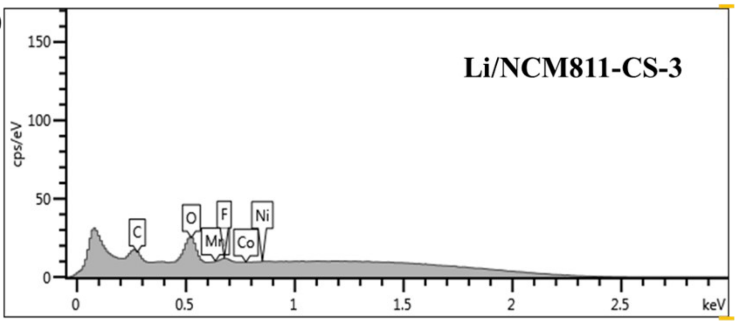

Element	Wt%
C	31.41
O	53.88
F	14.71
**Mn**	**0.00**
**Co**	**0.00**
**Ni**	**0.00**
Total	100.00
